# miR-34a-5p functions as a tumor suppressor in head and neck squamous cell cancer progression by targeting Flotillin-2

**DOI:** 10.7150/ijbs.64851

**Published:** 2021-10-21

**Authors:** Xiang Li, Shouwei Zhao, Yu Fu, Ping Zhang, Zhenxing Zhang, Jie Cheng, Laikui Liu, Hongbing Jiang

**Affiliations:** 1 Department of Oral and Maxillofacial Surgery, Affiliated Hospital of Stomatology, Nanjing Medical University, 136 Hanzhong Road, Nanjing 210029, Jiangsu Province, China.; 2 Jiangsu Key Laboratory of Oral Diseases, Nanjing Medical University, 136 Hanzhong Road, Nanjing 210029, Jiangsu Province, China.; 3 Jiangsu Province Engineering Research Center of Stomatological Translational Medicine.

**Keywords:** Head and Neck Squamous Cell Carcinoma, miR-34a-5p, FLOT-2, MEK/ERK1/2

## Abstract

While a number of therapeutic advances have been made in recent years, the overall survival of patients with head and neck squamous cell cancer (HNSCC) remains poor. MicroRNAs (miRNAs) are key drivers of oncogenic progression, with miR-34a-5p downregulation having been observed in many different tumor types. Here, we assessed the link between miR-34a-5p and HNSCC progression and the mechanistic basis for this relationship. Levels of miR-34a-5p in HNSCC tumors and cell lines were assessed via qPCR, after which we explored the functional importance of this miRNA in this oncogenic setting. Through luciferase reporter assays, the ability of miR-34a-5p to regulate flotillin-2 (FLOT-2) was further clarified. Overall, these analyses revealed that HNSCC tumors and cells exhibited marked miR-34a-5p downregulation that was linked to the progression of this tumor type. At a functional level, miR-34a-5p constrained the proliferation, migratory/invasive activity, and epithelial-mesenchymal transition induction in HNSCC cells. At the mechanistic level, miR-34a-5p was found to suppress FLOT-2 expression and to activate the MEK/ERK1/2 pathway. Overall, these results suggest that miR-34a-5p can function as a tumor suppressor miRNA in HNSCC owing to its ability to target FLOT-2, highlighting the promise of targeting this regulatory axis to treat HNSCC.

## Introduction

Head and neck squamous cell carcinoma (HNSCC) is the 6^th^ most prevalent form of cancer globally, and these tumors frequently undergo lymphatic metastasis such that the prognosis of patients is often very poor 1. As of 2019, there were an estimated 740,000 HNSCC cases diagnosed per year, with a 40-50% mortality rate for affected individuals. While there have been major recent advances in surgical techniques and other treatments for affected patients, the overall survival of this population has failed to improve substantially 2. These high mortality rates underscore the need to define and clinically apply novel biomarkers capable of predicting HNSCC onset or progression in order to improve the prognosis of individuals affected by this cancer.

MicroRNAs (miRNAs) are non-coding transcripts that are able to suppress the translation of specific complementary target mRNAs by binding to their 3'-UTR 3. In addition to this classical inhibitory mechanism, more recent work also suggests that certain miRNAs can control gene expression via binding to coding sequence (CDS) and 5'-UTR regions. The targeting of 3'-UTR sequences is more closely tied to mRNA degradation, whereas CDS targeting can suppress translation 4. MiR-34a-5p was recently identified as a miRNA that was potently upregulated by the tumor suppressor protein p53 5-7, functioning as a potent tumor suppressor miRNA in a variety of oncogenic contexts 8-10. There is also growing evidence that miR-34a can, conversely, function as an oncogenic mediator in certain brain tumors in which it is overexpressed 11. The roles played by miR-34a-5p in HNSCC, however, remain to be fully defined.

Flotillin-2 (FLOT-2) is a lipid raft component located in the cytoplasm and it is also associated with certain intracellular vesicles 12. Prior work has highlighted roles for FLOT-2 as a regulator of malignant processes including signal transduction, cellular adhesion, and protein trafficking 13. For example, in hepatocellular carcinoma (HCC), FLOT-2 was found to promote metastatic progression by controlling cell cycle progression and inducing the epithelial-mesenchymal transition (EMT) via MEK/Raf/ERK signaling activity 14. Liu et al. further demonstrated the ability of FLOT-2 to promote NF-κB and PI3K/Akt3 pathway activation and consequent metastatic progression in nasopharyngeal carcinoma (NPC) 15. A recent analysis also revealed that FLOT-2 can serve as a biomarker of lymphatic metastasis in NPC 16, in addition to being independently associated with HNSCC patient prognosis 17. The specific mechanisms whereby FLOT-2 influences HNSCC progression, however, remain to be defined.

Herein, we determined that miR-34a-5p was able to suppress HNSCC cell proliferation, EMT induction, and migratory/invasive activity by suppressing FLOT-2 expression and modulating MEK/ERK1/2 signaling activity. Together, these data highlight miR-34a-5p/FLOT-2 axis as a viable therapeutic target for this cancer type.

## Materials and Methods

### Patient samples

HNSCC patient tumor tissues and matched paracancerous samples collected ~5 cm from the tumor tissue were collected from 44 HNSC patients undergoing surgical treatment at the Department of Oral Pathology, School of Stomatology, Nanjing Medical University. Two experienced pathologists diagnosed all samples, with recruited patient information being detailed in Table S1. Samples were snap-frozen upon collection and stored at -80°C. Patients had not undergone local or systemic treatment prior to sample collection. Broder's classification system was used to classify tumor histological grade, while the TNM criteria from the International Union Against Cancer were used for tumor staging. All patients provided written informed consent, and the Research Ethics Committee of Nanjing Medical University approved this study, which was consistent with the Declaration of Helsinki.

### Cell culture

The human HN4 cell line was obtained from the Cell Bank of the Chinese Academy of Sciences (Shanghai, China), while HSC3, HN6, and CAL27 cells were from American Type Culture Collection (ATCC). All tumor cells were grown in DMEM/F12 media (Gibco, USA) supplemented with 10% FBS (HyClone, USA) and penicillin/streptomycin (Invitrogen, USA). HOK cells were grown in Oral Keratinocyte Medium (OKM, ScienCell, USA) containing 10% FBS and penicillin/streptomycin. All cells were grown in a humidified 5% CO_2_ incubator at 37°C, and had been passaged for < 6 months at the time of experimental use.

### Quantitative real-time PCR

TRIzol (Invitrogen, CA, USA) was used to extract cellular RNA based on provided directions. To measure FLOT-2 mRNA levels, a SYBR Premix ExTaq Reverse Transcription PCR kit (Takaka, Dalian, China) was used based on provided directions to conduct qPCR analyses, with *GAPDH* as a normalization control. TaqMan assay kits (Applied Biosystems, CA, USA) were used utilized to assess miR-34a-5p expression, with* U6* as a normalization control. All qPCR primers are listed in Table S2, and all assays were conducted with an ABI7900 instrument (Applied Biosystems), with the 2^-∆∆Ct^ method being utilized to assess relative gene expression 18.

### Cell transfection

All miR-34a-5p inhibitor, mimic, and negative control constructs, as well as siRNAs specific for FLOT-2 (5'-GCAGAGAGAUGCUGACAUUTT-3') or corresponding control siRNAs, were purchased from Genepharma (Shanghai, China). Cells were grown to 60% confluency in 6-well plates, and were transfected using Lipofectamine 2000 (Invitrogen, USA) based on provided directions. At 24 or 48 h post-transfection, RNA and protein were analyzed for downstream qRT-PCR and Western blotting analyses.

### Western blotting

RIPA buffer was used to extract total protein from cells for 30 min, after which protein was separated via SDS-PAGE and transferred onto an Immobilon-PVDF membrane (Millipore Corporation, MA, USA). Blots were blocked for 2 h with 5% non-fat milk prior to overnight incubation with appropriate primary antibodies at 4°C overnight. Blots were then rinsed with TBS-T and probed with secondary peroxidase-conjugated antibodies prior to protein band visualization with a chemiluminescence reagent (Millipore Corporation). Antibodies used for this study were specific for E-cadherin (#3195, CST), N-cadherin # (22018-1-AP, Proteintech), FLOT-2 (#28208-1-AP, Proteintech), Vimentin (#5741, CST), MEK (#11049-1-AP, Proteintech), p-MEK (#28930-1-AP, Proteintech), p-ERK (#AF1015, Affinity), ERK (#67170-1-Ig, Proteintech), CCND1 (#BF0127, Affinity) CDK4 (#DF6102, Affinity), CDK6 (#DF6448, Affinity).

### CCK-8 assay

A CCK-8 kit (Dojindo, Kumamoto, Japan) was used based on provided directions to assess cellular proliferation. Briefly, at 24 h post-transfection, cells were added to 96-well plates (3x10^3^/well). At appropriate time points, CCK-8 reagent was added to each well (10 μl), followed by an additional 2 h incubation at 37℃. Absorbance at 450 nm was then measured via microplate reader (Molecular Devices, Sunnyvale, USA).

### Wound-healing and invasion assay

Wound healing assays were conducted by growing CAL27 and HN4 cells to 90% confluence in 6-well plates, at which time a scratch wound was generated in the monolayer surface with a sterile micropipette tip. Debris was then washed away, and remaining cells were cultured in serum-free media, with wound closure being measured at appropriate timepoints as a correlate for migration. To assess invasion, at 24 h post-transfection, 5x10^4^ cells in serum-free media were added to the upper chamber of a Transwell insert (6.5 mm diameter, 8-μm pore size, Millipore Corporation). The lower chamber was then filled with DMEM/F12 supplemented with 10% FBS. Following a 24 or 36 h incubation, cells were fixed with paraformaldehyde (PFA), stained using crystal violet, and imaged in three randomly selected fields of view per well.

### Dual-luciferase reporter assay

Targetscan was used to identify a putative miR-34a-5p binding site in FLOT-2, after which wild-type (WT) and mutant versions of this FLOT-2-3'UTR sequence were generated and separately cloned into the pEZX-FR02 vector (Genecopoeia, USA). Cells were then transfected with these reporter plasmids and miR-34a-5p mimics using Lipofectamine 2000. At 48 h post-transfection, a Dual Luciferase Assay kit (Promega, WI, USA) was used based on provided directions.

### Immunohistochemistry (IHC) staining

Paraffin-embedded tissue sections (5 μm) were heated overnight at 37°C. Xylene was used to deparaffinize these samples, after which they were hydrated using an ethanol gradient. Samples were then heated with citrate buffer for 15 min in a pressure cooker. Next, 3% H_2_O_2_ was used to treat samples for 20 min at room temperature to block endogenous peroxidase activity. After overnight staining with appropriate primary antibodies, 3,3-diaminobenzidine (DAB) was then used for color development, followed by hematoxylin counterstaining for 10 min. For negative control staining, PBS was used in place of the primary antibody. Samples were evaluated based on both staining intensity and the degree of staining. Staining intensity was scored as: 0 (Negative), 1 (Weak), and 2 (Strong). Degree of staining was scored based on the percentage of brown-stained HNSCC cells as: 0 (0%), 1 (1-50%), and 2 (51-100%). Overall staining scores were calculated by multiplying these two scores together, with an overall score of 0-1 being considered indicative of low expression, while scores of 2-4 were indicative of high expression.

### Animal experiments

The Committee on Animal Ethics of Nanjing Medical University approved all animal studies, which were consistent with laboratory animal welfare protocols. Briefly, BALB/c nude mice (4-weeks-old) from the Animal Core Facility of Nanjing Medical University were subcutaneously implanted in the flank with 2×10^7^ CAL27 cells in Matrigel. On day 20 post-implantation, 5 nmol of AgomiRNA-34a-5p or negative control was intratumorally injected into these animals every 5 days, with 5 total injections. Tumor width and length were assessed every four days. On day 40, mice were euthanized and tumor samples were collected for analysis. Tumor volume (mm^3^) = Tumor width (mm)^2^ × tumor length (mm)/2.

### Statistics analysis

GraphPad Prism 8 was used to analyze all data, which are given as means ± standard error. Paired samples t-tests were used to compare miR-34a-5p expression levels between HNSCC tumors and paracancerous samples. Independent samples t-tests were used to assess differences between high/low histological grades, high/low stages, and transfection-related changes in protein expression, gene expression, and invasive/migratory activity. Survival outcomes were assessed with log-rank tests. A two-sided P < 0.05 was the significance threshold.

## Results

### miR-34a-5p downregulation in HNSCC tumors is linked with disease progression

**Figure 1:**
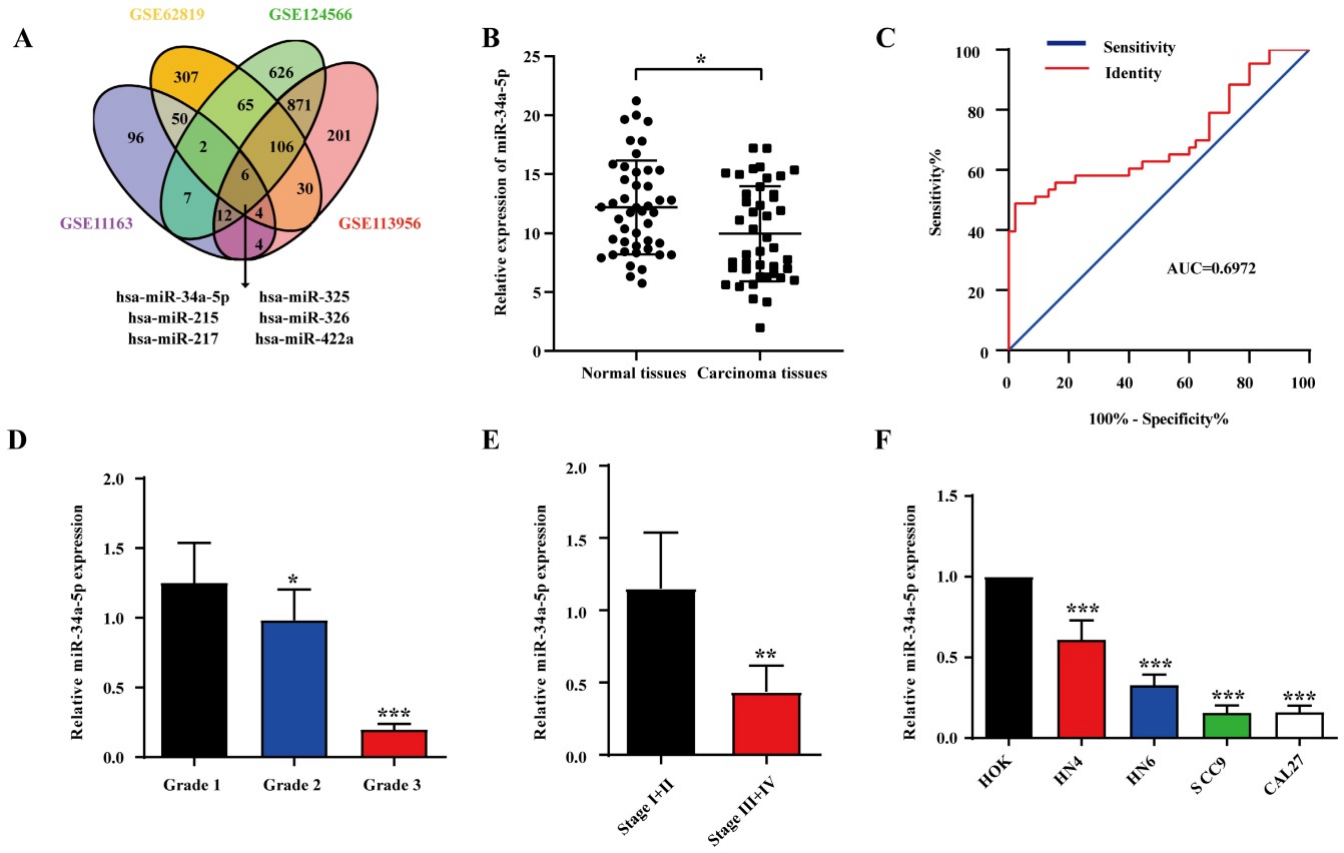
** miR-34a-5p downregulation in HNSCC is associated with tumor progression. A.** Venn diagram of HNSCC-related miRNAs in GSE1163, GSE62819, GSE124566 and GSE113596. Thereinto 6 downregulated expressed miRNAs were listed.** B.** The expression of miR-34a-5p in tumor and adjacent normal tissue from patients with HNSCC was analyzed by qRT-PCR (n = 44,* p* < 0.05). **C.** ROC curves showing the diagnostic performance of miR-34a-5p in HNSCC (AUC indicates the area under ROC curve). **D.** Low expression of miR-34a-5p was significantly correlated with poor tumor differentiation. **E.** Low expression of miR-34a-5p was significantly correlated with advanced clinic stage.** F.** The expression level of miR-34a-5p was significantly lower in HNSCC cell lines compared with HOK. Data were presented as the mean ± SEM from three independent experiments. *p < 0.05; **p < 0.01; ***p<0.001.

To explore miRNA expression patterns in HNSCC, we identified four relevant datasets (GSE11163, GSE62819, GSE124566, and GSE113956; Table 1). In total, 6 miRNAs were analyzed in each platform (Fig. 1A), revealing miR-34a-5p downregulation, which was considered noteworthy given its previously documented relationship with other human cancer types. To explore miR-34a-5p expression levels in further detail, we analyzed HNSCC patient tumors and paracancerous tissues via qPCR. Details pertaining to these patients are compiled in Table 2. Significant miR-34a-5p downregulation was evident in HNSCC tumors relative to adjacent normal tissues (Fig. 1B). To explore the diagnostic value of this miRNA in the context of HNSCC, ROC curves were constructed using these qPCR results. The area under the ROC curve (AUC) value for this analysis was 0.6972 (Fig. 1C), indicating that miR-34a-5p levels can be utilized to differentiate between HNSCC tumors and normal tissues. We additionally observed a correlation between low miR-34a-5p levels and both poor tumor differentiation (Fig. 1D) and advanced TNM stage (Fig. 1E). Additional qPCR analyses of HNSCC cell lines revealed the significant downregulation of this miRNA in tested cell lines relative to control human oral epithelial keratinocyte (HOK) cells (Fig. 1F). The downregulation of miR-34a-5p was thus correlated with more advanced HNSCC progression.

### miR-34a-5p inhibits EMT induction as well as migratory, proliferative, and invasive activity in HNSCC

**Figure 2:**
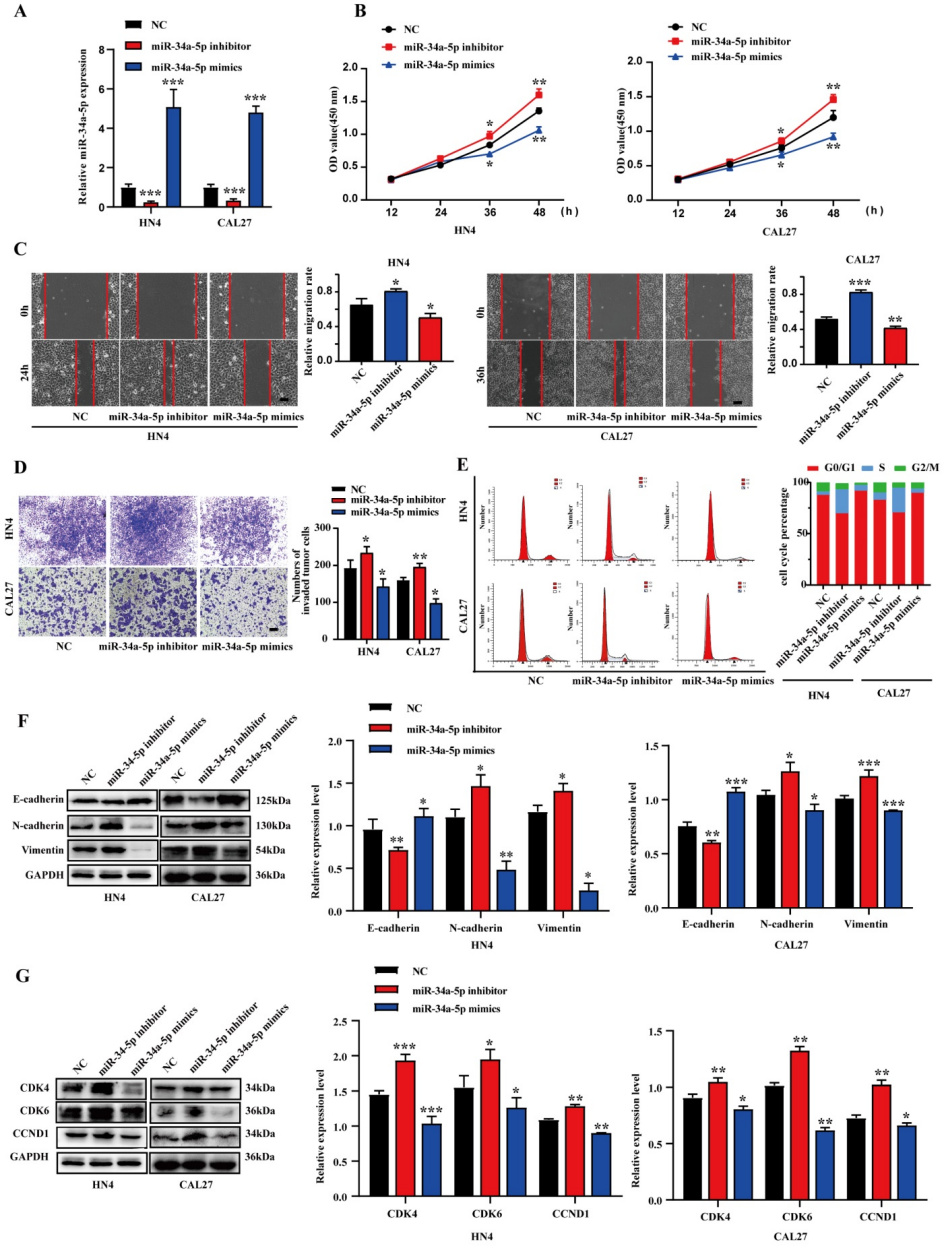
** miR-34a-5p could suppress the proliferation and progression of HNSCC cells. A**. qRT-PCR analysis revealed the effects of miR-34a-5p mimics and miR-34a-5p inhibitors on miR-34a-5p expression in HN4 and CAL27 cells. **B.** HN4 and CAL27 cells were transfected with miR-34a-5p mimics or inhibitor, and proliferation ability of cells was detected by CCK8 assay. **C.** Wound healing assay showed that overexpression or downregulation of miR-34a-5p decreased or increased the cell migration ability.** D.** Invasive ability of HN4 and CAL27 cells transfected with mimics or inhibitor were analyzed using Transwell chamber assay. **E.** Cell cycle distribution in HN4 and CAL27 cells tranfected with miR-34a-5p inhibitor or mimics was analyzed by flow cytometry.** F and G.** Protein expression levels of EMT (**F**) and cell cycle regulators (**G**) markers were detected via western blot analysis in HN4 and CAL27 cells. Data were presented as the mean ± SEM from three independent experiments. *p < 0.05; **p < 0.01; ***p<0.001.

To evaluate the ability of miR-34a-5p to suppress HNSCC progression, miR-34a-5p inhibitors or mimics were transfected into CAL27 or HN4 cells (Fig. 2A). Consistent with our expectations, miR-34a-5p inhibition enhanced the proliferative activity of tumor cells, whereas miR-34a-5p mimic transfection had the opposite effect (Fig. 2B). In a wound healing assay, miR-34a-5p inhibition was associated with enhanced migratory activity, while the overexpression of this miRNA impaired HNSCC cell migration (Fig. 2C). Similarly, the invasive activity of these HNSCC cell lines was respectively impaired and enhanced by miR-34a-5p mimic and inhibitor transfection in a Transwell assay system (Fig. 2D). Given the observed changes in cell proliferation, a cell cycle analysis was next conducted via flow cytometry. In this analysis, miR-34a-5p mimic transfection was associated with an increase in the frequency of cells in the G0/G1 stage relative to control (92.64% vs. 86.95% in HN4, 66.17% vs. 63.02% in CAL27), while miR-34a-5p inhibition decreased this frequency from 86.95% to 71.49% and 63.02% to 59.48% in HN4 and CAL27, respectively (Fig. 2E). These results were also confirmed through Western blotting analyses of cell cycle regulatory proteins. Increased E-cadherin levels together with reductions in Vimentin and N-cadherin were also observed in these cell lines following miR-34a-5p mimic transfection, while the opposite was observed following the inhibition of this miRNA (Fig. 2F). Together, these results indicate that miR-34a-5p can suppress HNSCC cell migratory, proliferative, and EMT activity *in vitro.*

### FLOT-2 is a miR-34a-5p target gene in HNSCC

**Figure 3:**
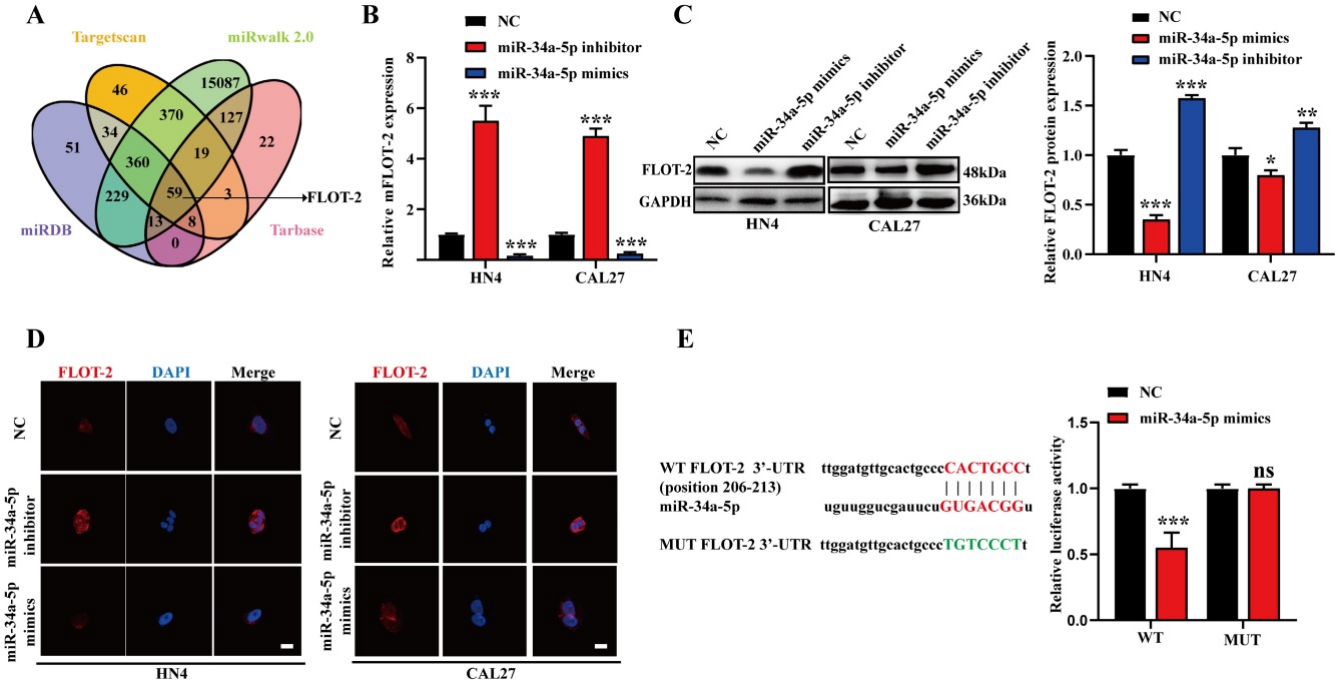
**FLOT-2 is a direct target of miR-34a in HNSCC. A.** Combined analysis of bioinformatics prediction to screen for miR-34a-5p-binding mRNAs. **B.** The FLOT-2 mRNA expression in HN4 and CAL27 cells with miR-34a-5p inhibitor or mimics transfection was detect by qRT-PCR. **C, D.** The FLOT-2 protein expression in HN4 and CAL27 cells with miR-34a-5p inhibitor or mimics transfection was detect by western blot analysis and immunofluorescence staining. **E.** The effect of miR-181a-5p on a dual-luciferase reporter plasmid bearing wild-type (WT)/mutated (MUT) FLOT-2 binding sites was analyzed. Data were presented as the mean ± SEM from three independent experiments.*p < 0.05; **p < 0.01; ***p<0.001; ns P > 0.05.

Putative miR-34a-5p target genes were next identified with the Targetscan, miRwalk, and miRDB databases, revealing Watson-Crick complementarity between this miRNA and the FLOT-2 3'-UTR (Fig. 3A), thus suggesting miR-34a-5p has the potential to downregulate this gene. To test this possibility, we transfected CAL27 and HN4 cells with miR-34a-5p inhibitor or mimic constructs, and found that this resulted in respective increases and decreases in FLOT-2 expression at the mRNA and protein levels (Fig. 3B-D). To formally test the ability of these two targets to directly interact with one another, we generated luciferase reporter constructs encoding the WT or mutant (MUT) version of the predicted miR-34a-5p binding sequence in the FLOT-2 3'-UTR and then transfected these vectors into HEK293T cells along with miR-34a-5p mimics or negative control constructs. Luciferase activity was decreased following miR-34a-5p mimic and WT but not MUT reporter plasmid transfection (Fig. 3E), thus confirming that FLOT-2 is a direct miR-34a-5p target gene, suggesting a potential pathway whereby this miRNA may regulate the progression of HNSCC.

### FLOT-2 silencing suppresses HNSCC cell malignancy

**Figure 4:**
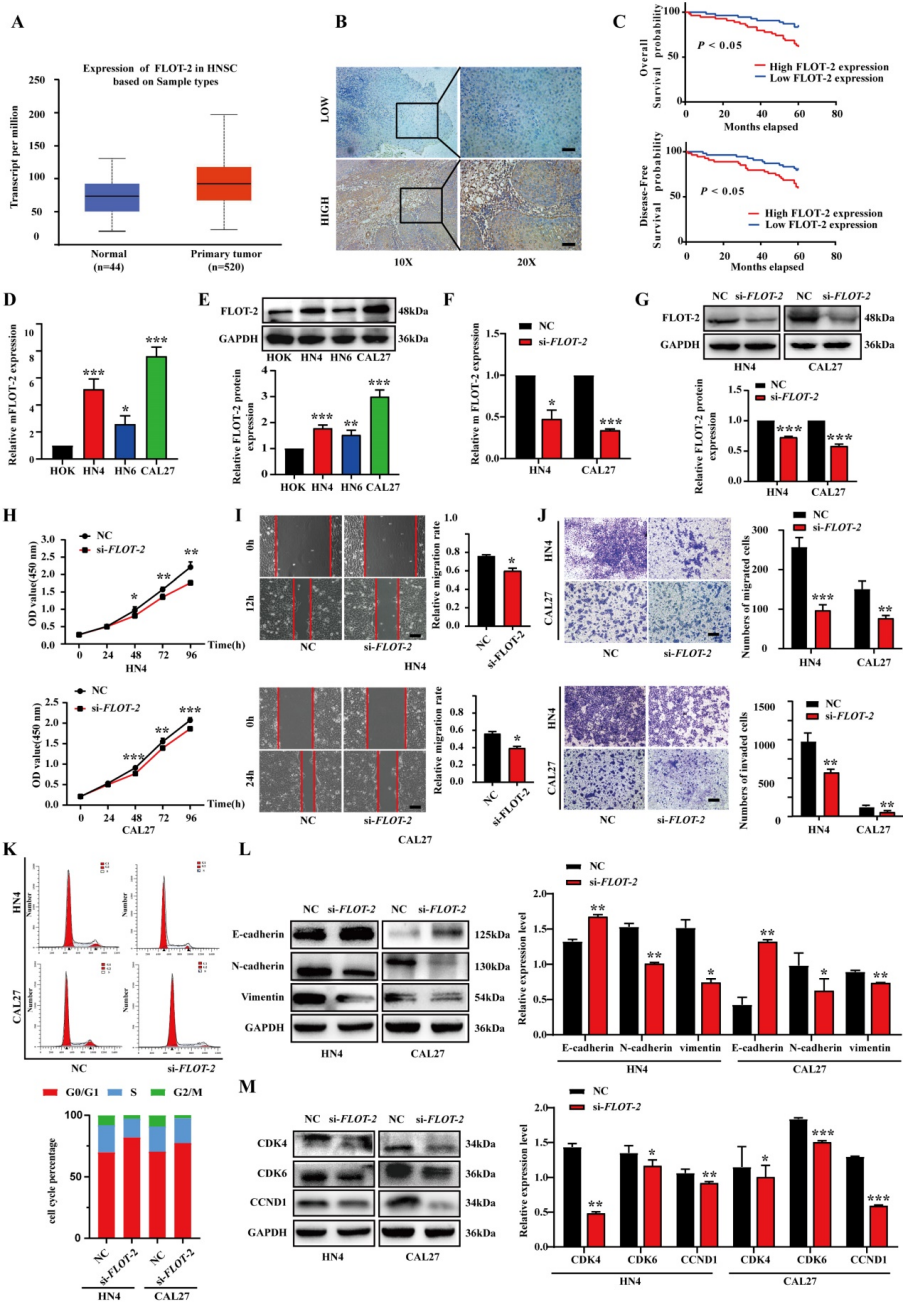
** Downregulation of FLOT-2 inhibited the proliferation, migratory, invasive activity and cell cycle in HN4 and CAL27 cells. A.** Expression of FLOT-2 mRNAs in primary tumor and normal samples in the TCGA HNSCC database. **B.** Low and high expression of FLOT-2 protein in HNSCC tissues were detected by IHC. **C.** Kaplan-Meier analysis to plot the overall survival curves and disease-free survival curves of HNSCC patients with expression of FLOT-2 and different clinicopathological characteristics and statistical significance was assessed, and it is indicated that high expression of FLOT-2 was associated with the poor prognosis and disease-free survival. **D and E.** Real-time RT-PCR assay and western blotting analyses were performed to detect the expression of FLOT-2 at the mRNA and protein levels, in HOK, HN4, HN6 and CAL27 cells, respectively. **F and G.** Real-time RT-PCR and western blotting analyses were performed to measure the expression of FLOT-2 in HN4 and CAL27 cells after FLOT-2 siRNA transfection, respectively.** H.** HN4 and CAL27 were transfection with si-*FLOT-2*, and the proliferation ability of cells was detected by CCK8 assay. **I and J.** Migrative and invasive ability of HN4 and CAL27 cells transfected with si-*FLOT-2* were analyzed using wound healing assay and transwell chamber assay. **K.** Cell cycle distribution was analyzed by flow cytometry. **L and M.** Western blot analysis was used to detect protein expression levels of EMT (**L**) and cell cycle regulators (**M**) markers in HN4 and CAL27 cells. Data were presented as the mean ± SEM from three independent experiments. *p < 0.05; **p < 0.01; ***p<0.001.

To evaluate the functional importance of FLOT-2 in HNSCC, we next evaluated the expression of this protein in this cancer type using the TCGA database (Fig. 4A), categorizing HNSCC tissues into FLOT-2-high or -low samples based on immunostaining scores (Fig. 4B). When we evaluated the relationship between FLOT-2 expression and patient survival, we found that higher FLOT-2 expression was associated with worse overall survival (OS) and disease‑free survival (DFS) (*p*<0.05) (Fig. 4C). Similarly, FLOT-2 mRNA and protein levels were upregulated in HN4, HN6, and CAL27 cells relative to HOK cells (Fig. 4D-E). In addition, FLOT-2 mRNA levels in HN4 and CAL27 cells were over 5-fold increased relative to HOK cells. When FLOT-2 was knocked down using siRNA constructs (*si-FLOT-2*), we observed significant decreases in FLOT-2 protein and mRNA levels, as expected (Fig. 4F-G). FLOT-2 silencing suppressed HNSCC cell proliferation in a CCK-8 assay (Fig. 4H), and similarly impaired the migratory and invasive activities of these cells in wound healing and Transwell assays (Fig. 4I-J). Moreover, the frequency of HN4 and CAL27 cells in the G0/G1 phase of the cell cycle was increased following si-FLOT-2 transfection (82.64% and 77.45%, respectively) relative to control cells (71.49% and 70.54%, respectively) (Fig. 4K). Such FLOT-2 knockdown was associated with elevated E-cadherin and reduced N-cadherin and Vimentin expression in both cell lines, consistent with EMT suppression (Fig. 4L). Although conflicting results have been published regarding the effects of cyclin D1^19^, reductions in CDK4, CDK6, and CCND1 protein levels were observed in our results, consistent with G0/G1-phase cell cycle arrest (Fig. 4M). Overall, these findings indicated that reductions in the expression of FLOT-2 can suppress HNSCC cell migration, proliferation, and EMT induction.

### miR-34a-5p regulates HNSCC cell malignancy by targeting FLOT-2

**Figure 5:**
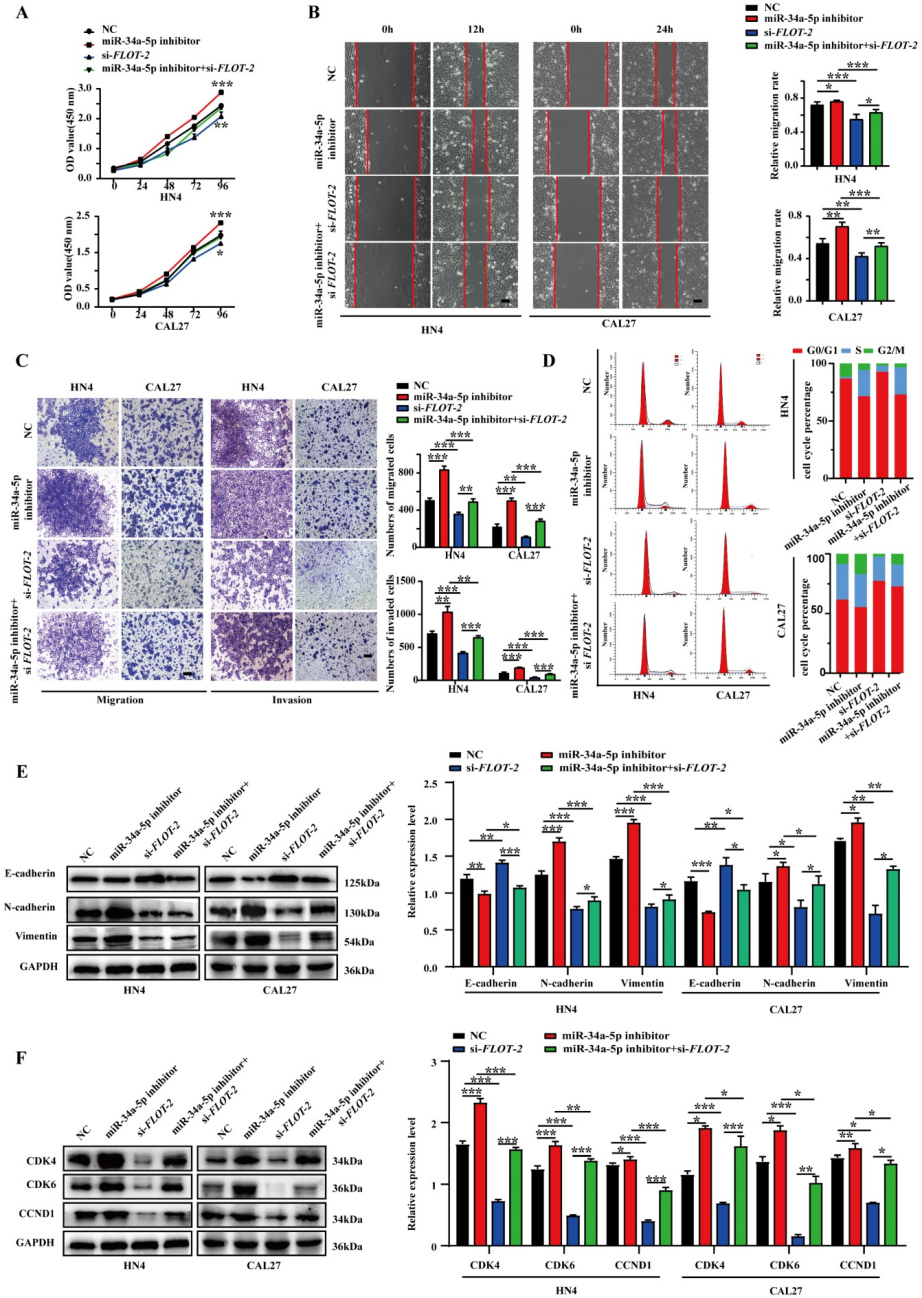
** FLOT-2 reversed the tumor-suppressing effect of miR-34a-5p in HNSCC cells. A**. HN4 and CAL27 cells were transfected with si-*FLOT-2*, miR-34a-5p inhibitor or co-transfected with si-*FLOT-2* and miR-34a-5p inhibitor. Cell proliferation was determined by CCK8 assay. **B.** Effects of si-*FLOT-2*, miR-34a-5p inhibitor or co-transfected with si-*FLOT-2* and miR-34a-5p inhibitor on the migration of HN4 and CAL27 cells were evaluated by wound healing assay and transwell migration assays. **C.** Transwell invasion assays were used to detected the invasion ability of HN4 and CAL27 cells transfected with si-*FLOT-2*, miR-34a-5p inhibitor or co-transfected with si-*FLOT-2* and miR-34a-5p inhibitor. **D.** Cell cycle analysis by flow cytometry revealed that si*-FLOT-2* raised the proportion of G0/G1-phase cells could be partly rescued by miR-34a-5p inhibitor in HN4 and CAL27 cells. **E and F.** Western blot analysis of EMT (**E**) and related cell cycle regulators (**F**) markers. Data were presented as the mean ± SEM from three independent experiments. *p < 0.05; **p < 0.01; ***p<0.001.

To confirm that miR-34a-5p-mediated FLOT-2 silencing plays a functional role in regulating the malignancy of HNSCC cells, we next conducted rescue experiments in which cells were transfected with miR-34a-5p inhibitors and si-*FLOT-2* and the phenotypes of these cells were evaluated. This co-transfection approach partially reversed the effects of both FLOT-2 knockdown and miR-34a-5p inhibition on cell proliferation (Fig. 5A). Moreover, miR-34a-5p inhibitor and si-*FLOT-2* transfection partially reversed the effects of these individual treatments on HNSCC cell migration and invasion (Fig. 5B-C). Similarly, relative to the frequency of cells in the G0/G1 phase following miR-34a-5p inhibitor or si-*FLOT-2* transfection (71.49% or 92.64% in HN4 and 59.48% or 77.45% in CAL27, respectively), co-transfection was sufficient to partially reverse these phenotypes (73.03% in HN4, 72.87% in CAL27). Interactions between miR-34a-5p and FLOT-2 were also confirmed in the context of EMT induction (Fig. 5E) and cell cycle-related protein expression (Fig. 5F). Together, these results indicated that miR-34a-5p inhibition can enhance the malignant properties of HNSCC cells, while simultaneous FLOT-2 knockdown was sufficient to partially reverse these oncogenic effects, consistent with a direct regulatory relationship between these two molecules in tumor cells.

### miR-34a-5p suppresses HNSCC cell growth by inhibiting the MEK/ERK1/2 pathway

**Figure 6:**
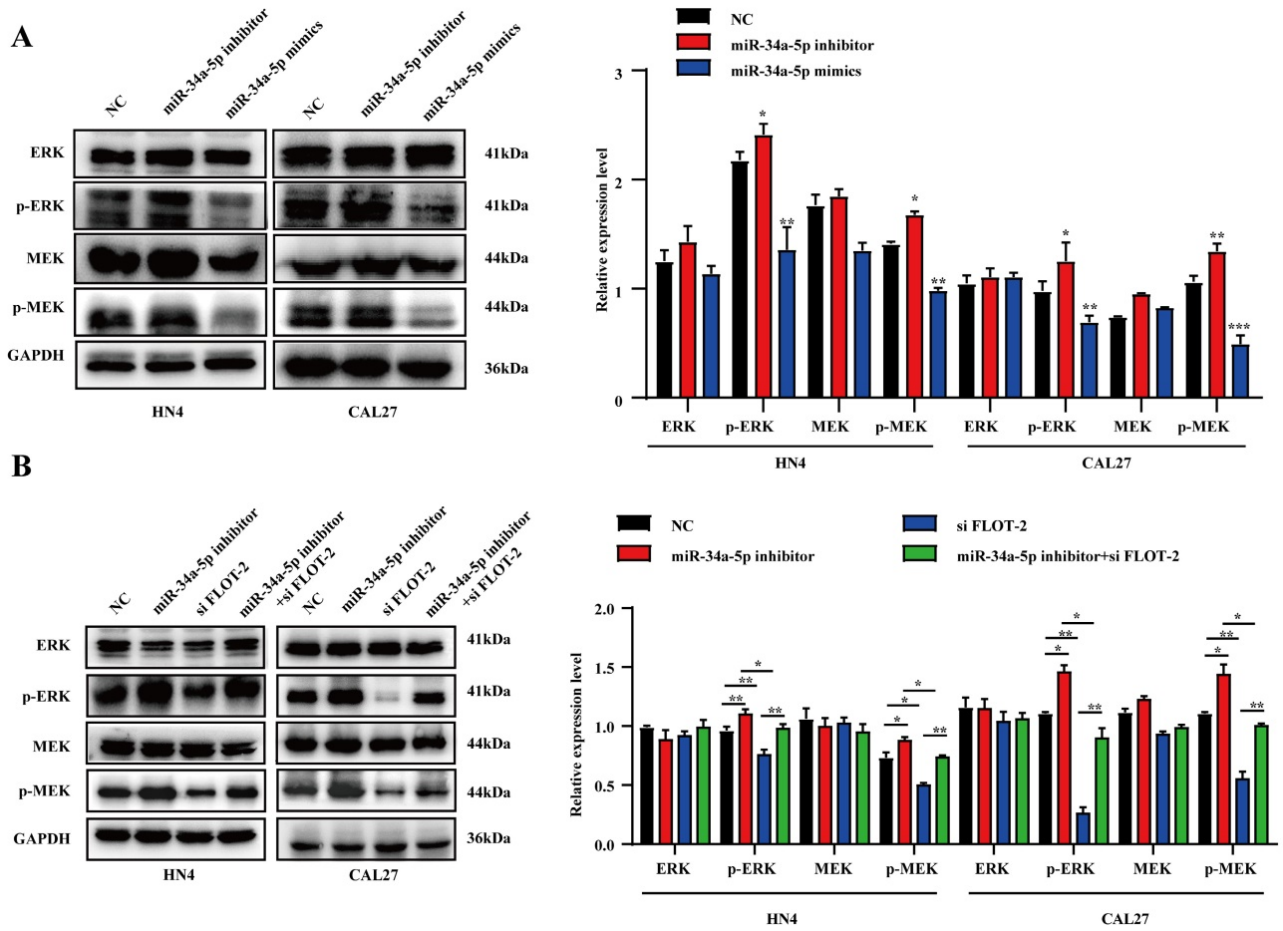
**miR-34a-5p inhibitor induces activation of the MEK/ERK1/2 signaling pathway in head and neck squamous cell carcinoma cells. A.** Protein expression levels were evaluated by western blotting in HNSCC cells transfected with miR-34a-5p inhibitor or mimics. **B.** Western blot analysis of p‐MEK, MEK, p‐ERK1/2, and ERK1/2 regulated by si-*FLOT-2*, miR-34a-5p inhibitor or co-transfected with si-*FLOT-2* and miR-34a-5p inhibitor. Data are presented as the mean ± SEM from three independent experiments. *p < 0.05; **p < 0.01; ***p<0.001.

To more fully explore the link between miR‐34a-5p, FLOT-2, and HNSCC, Western blotting was used to assess MEK/ERK1/2 pathway protein levels. MiR-34a-5p mimic transfection was associated with significant reductions in p‐MEK/MEK and p‐ERK1/2/ERK1/2 levels (Fig. 6A), whereas miR-34a-5p inhibitor treatment resulted in increases in these levels in both CAL27 and HN4 cells. Moreover, si-*FLOT-2* transfection reduced p‐MEK/MEK and p‐ERK1/2/ERK1/2 levels, while combined si*-FLOT-2* and miR‐34a-5p inhibitor transfection partially rescued these levels (Fig. 6B, *p* < 0.05). These results thus supported a model wherein miR-34a-5p was able to regulate FLOT-2 and thereby modulate MEK/ERK/1/2 pathway activity within HNSCC cells.

### miR-34a-5p treatment suppresses *in vivo* HNSCC tumor malignancy

**Figure 7:**
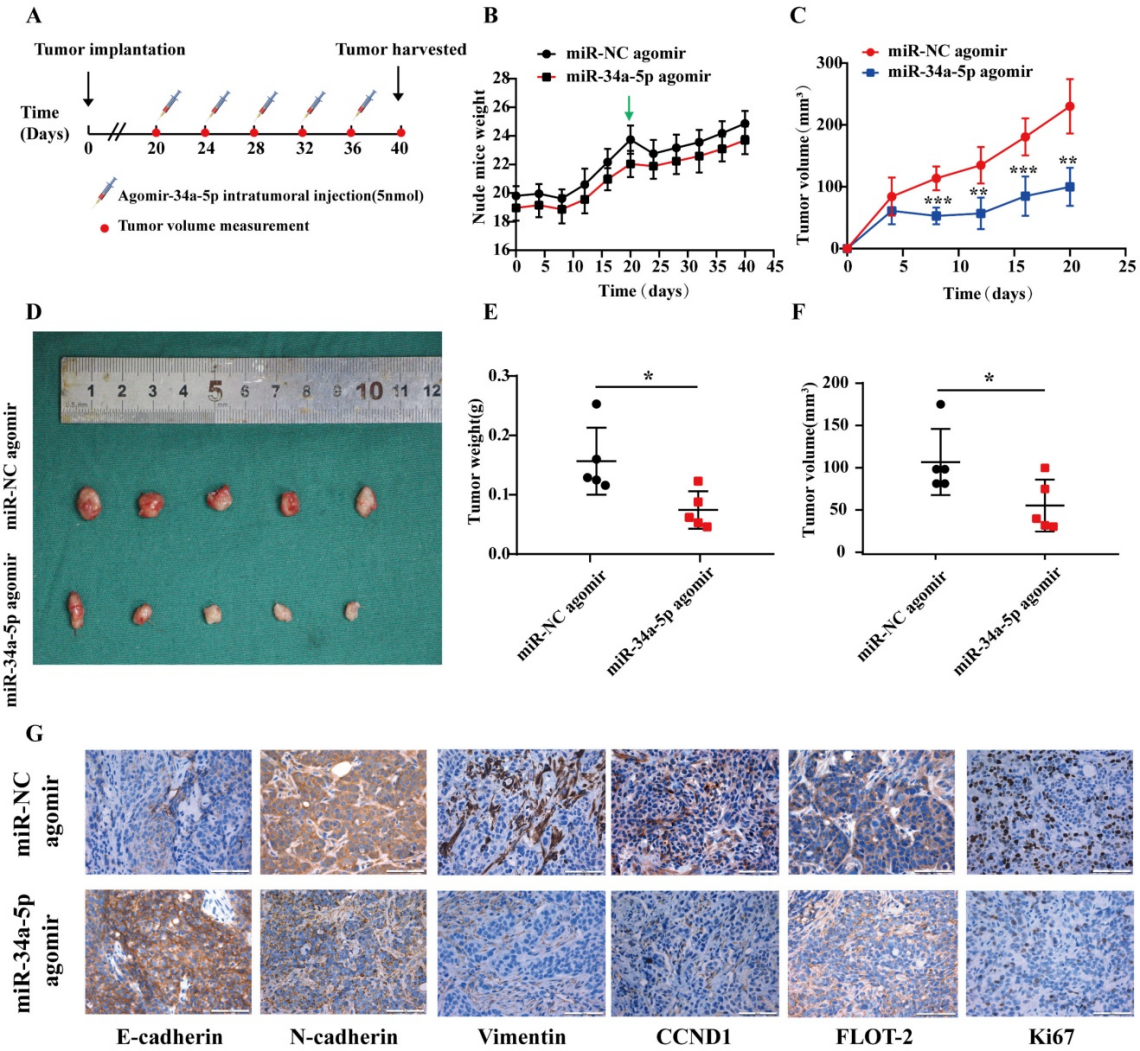
** miRNA-34a-5p agomir represses tumor growth of CAL27 cell in xenograft model. A.** CAL27 cells were subcutaneously injected into flanks of BALB/c nude mice (5 nmol, intratumoral injection, every 4 days). n= 5 per group. **B and C.** Weight of nude mice (**B**) and tumor volume (**C**) were measured every 4 days. Green arrows indicate the start of treatment. **D.** Tumor volume images. MiR-34a-5p agomir inhibited tumor growth *in vivo*.** E and F.** Scatter plot of xenograft tumor weight (**E**) and tumor volume (**F**). **G.** Representative micrographs showing IHC staining of E-cadherin, N-cadherin, Vimentin, CCND1, FLOT-2 and Ki67 proteins in mouse tumor tissues. Representative staining images are shown. Scale bars = 50 μm.

Finally, we evaluated the suppressive activity of miR-34a-5p *in vivo* using a xenograft model system in which CAL27 cells were subcutaneously implanted into the flanks of nude mice, and agomiR-34a-5p (5 nmol) or corresponding control was injected into the tumors beginning on day 20 with 5 total injections per mouse (Fig. 7A). AgomiR-34a-5p was associated with a reduction in tumor volume relative to control treatment without any corresponding drop in murine body weight (Fig. 7B-F). Consistent with the above results, IHC staining of tumors from these animals revealed that agomiR-34a-5p treatment was associated with reductions in Vimentin, N-cadherin, FLOT-2, CCND1, and Ki67 protein expression (Fig. 7G).

## Discussion

While there have been major advances in current understanding of the mechanistic basis for HNSCC in recent years, the prognosis of affected patients remains poor 20. Prognostic evaluations of patients have the potential to guide more accurate treatment in an effort to enhance patient survival outcomes, making it essential that the mechanisms governing HNSCC be more fully clarified in order to facilitate such evaluations. Functionally, miRNAs can interact with Dicer and RISC to target complementary mRNAs, thereby disrupting their translation or promoting their degradation 21. Key miRNAs previously shown to suppress HNSCC growth including miR-410, miR-433, miR-329, and miR-195-5p 22-24. How miR-34a-5p impacts HNSCC, however, has not been explored in prior studies. Herein, we detected miR-34a-5p downregulation in HNSCC tissues and cells, and found such downregulation to be correlated with advanced TNM stage and poor tumor differentiation. Mechanistically, this miRNA was able to suppress HNSCC cell migratory, proliferative, and EMT activity.

Identifying miRNA target genes is vital to understanding how they affect oncogenic processes. Subsequent bioinformatics analyses revealed the ability of miR-34a-5p to bind to a complementary sequence in FLOT-2, suggesting a potential mechanism whereby this miRNA may influence oncogenic processes. When miR-34a-5p was overexpressed in four HNSCC cell lines, this was associated with FLOT-2 knockdown at the mRNA and protein levels. Moreover, miR-34a-5p was able to regulate a luciferase reporter harboring a WT but not mutant version of the FLOT-2 3'-UTR, consistent with the ability of this miRNA to directly suppress FLOT-2 expression. This study is the first to our knowledge to demonstrate the ability of miR-34a-5p to suppress HNSCC oncogenesis via targeting FLOT-2.

FLOT-2 is a scaffold protein associated with lipid rafts that is composed of highly ordered subdomains and plays essential roles in modulating the ability of these rafts to influence a range of biological processes. Prior work has linked FLOT-2 to poor survival and enhanced progression and metastasis in a range of cancers 16, 17, 25. The role of FLOT-2 in HNSCC, however, has not been previously clarified. Herein, an IHC staining analysis revealed FLOT-2 upregulation to be associated with a poorer prognosis. Higher levels of FLOT-2 expression were also able to promote HNSCC cell proliferation, cell cycle progression, and EMT induction, potentially contributing to negative outcomes. These data highlight the promise of FLOT-2 as a key regulator of HNSCC progression.

The MEK/ERK1/2 pathway is a key mediator of cellular proliferative, migratory, and invasive activity that is commonly activated in oncogenic settings including gallbladder cancer, breast cancer, and HNSCC 26-28. Several different miRNAs have been shown to regulate this signaling axis in various cancers. In one report, for example, Liu et al. demonstrated the ability of miR-34a/c-induced caprine endometrial epithelial cell apoptosis to regulate circ-8073/CEP55 through the RAS/RAF/MEK/ERK and PI3K/AKT/mTOR signaling pathways 29. Wang et al. further demonstrated in NSCLC that miR-760 could suppress tumor progression via disrupting ROS1/Ras/Raf/MEK/ERK pathway activation 30. Herein, we found that the downregulation of miR-34a-5p suppressed HNSCC malignancy by modulating the MEK/ERK1/2 pathway, with FLOT-2 downregulation being sufficient to partially reverse this effect, thus highlighting a novel mechanism whereby this miRNA can regulate oncogenesis.

**Figure 8:**
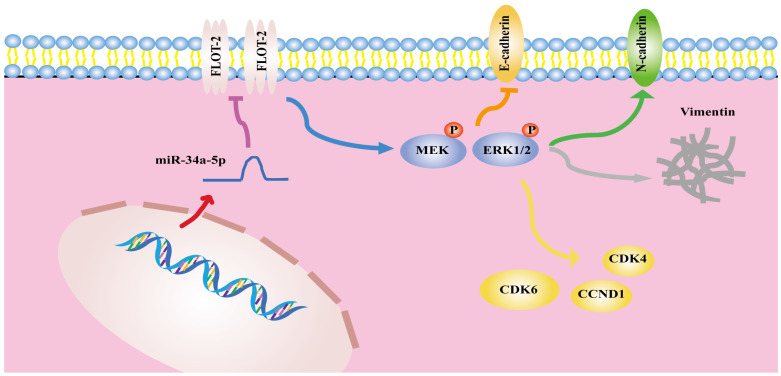
The mechanism diagram of the relationship among miR-34a-5p, FLOT-2 and MEK/ERK1/2 pathway in head and neck squamous cell cancer (HNSCC).

In summary, the results of the present analysis highlighted miR-34a-5p as a novel mediator of antitumor activity in HSCCC that functions in part by suppressing FLOT-2 expression and disrupting MEK/ERK1/2 pathway activation, thereby impairing cell cycle progression, migration, and invasion (Figure 8). This miR-34a-5p-FLOT-2-MEK/ERK1/2 axis may thus represent a viable target for HNSCC treatment.

## Supplementary Material

Supplementary tables.

## Figures and Tables

**Table 1:** Summary of the datasets used in our study.

Study	Platform	Technology	Country	downregulated miRNA in platform	Sample	Cancer	Normal	HNSCC cell lines
GSE11163	GPL6690	microRNAs array	USA	181	23	16	5	2
GSE62819	GPL16384	microRNAs array	China	570	5	5	5	0
GSE124566	GPL18402	microRNAs array	China	1695	10	10	10	0
GSE113956	GPL18058	microRNAs array	China	1234	40	25	15	0

**Table 2:** Correlation between miR-34a-5p expression and clinicopathologic features in 44 patients with OSCC

Feature	miR-34a-5p		χ^2^	*P*
	Low	High		
All	22	22		
Age			2.7300	0.0985
>60	14	6		
≤60	8	16		
Gender			0.3761	0.5397
Male	12	14		
Female	10	8		
Pathologic Grade			4.6970	0.0302*
I	16	7		
II-III	6	15		
TNM Stage			7.3330	0.0068**
I-II	17	9		
III-IV	5	13		
