# Supplementary material for: miR-34a-5p functions as a tumor suppressor in head and neck squamous cell cancer progression by targeting Flotillin-2
Source: Int J Biol Sci. 2021 Oct 21;17(15):4327–39. doi: 10.7150/ijbs.64851 (PMC8579463; doi:10.7150/ijbs.64851)
Supplement: Supplementary file 1 — Supplementary tables. [file ijbsv17p4327s1.pdf]

Supplementary Materials

Table S1. Clinical features of 44 patients with HNSCC

NO.	Gender	Age	Location	T	N	M	Clinical stage	Pathological grade
1	female	71	Gingiva	1	0	0	I	I
2	female	91	Gingiva	1	0	0	I	I-II
3	male	77	Tongue	1	0	0	I	I
4	male	71	Gingiva	1	0	0	I	I
5	male	67	Palate	1	0	0	I	I-II
6	female	65	Tongue	1	0	0	I	I-II
7	male	60	Buccal	1	0	0	I	I
8	male	55	Oropharynx, tongue	1	0	0	I	I-II
9	male	51	Floor of mouth	1	0	0	I	I
10	male	51	Buccal	1	0	0	I	I-II
11	female	49	Tongue	1	0	0	I	I
12	male	46	Tongue	1	0	0	I	II
13	female	45	Buccal	1	0	0	I	I
14	female	77	Tongue	2	0	0	II	I
15	female	77	Gingiva	2	0	0	II	I
16	female	70	Gingiva	2	0	0	II	I
17	male	69	Buccal	2	0	0	II	I
18	female	69	Tongue	2	0	0	II	I
19	male	58	Floor of mouth	2	0	0	II	I
20	female	57	Buccal	2	0	0	II	I-II
21	male	56	Floor of mouth	2	0	0	II	I
22	male	53	Gingiva	2	0	0	II	I
23	male	53	Lip	2	0	0	II	I
24	male	46	Gingiva	2	0	0	II	I
25	male	45	Buccal	2	0	0	II	I-II
26	female	78	Palate	1	1	0	III	I-II
27	female	77	Buccal	1	1	0	III	I-II
28	male	64	Tongue	1	1	0	III	I-II
29	male	51	Tongue	1	1	0	III	I
30	female	50	Tongue	1	1	0	III	I
31	male	46	Gingiva	1	1	0	III	I-II
32	male	44	Tongue	1	1	0	III	II-III
33	female	31	Tongue	1	1	0	III	I
34	female	83	Tongue	2	1	0	III	I-II
35	female	71	Buccal	2	1	0	III	I-II
36	male	71	Buccal	2	1	0	III	I
37	male	65	Gingiva	2	1	0	III	I-II
38	male	64	Gingiva	2	1	0	I	I
39	male	61	Buccal	2	1	0	III	I-II

40	male	54	Floor of mouth, tongue	2	1	0	III	II-III
41	female	54	Tongue	2	1	0	III	I-II
42	female	54	Gingiva	2	1	0	III	I
43	male	54	Tongue, floor of mouth	2	1	0	III	I-II
44	male	47	Tongue	2	1	0	III	I-II

Table S2. Primers of qRT-PCR

Target Gene	Primer Sequences (5` -3`)
miR-34a-5p	5′-UGGCAGUGUCUUAGCUGGUUGU -3′
U6	5′-ATGGACTATCATATGCTTACCGTA-3′
FLOT-2	F:5′-GAGATTGAGATTGAGGTTGTG-3′
	R:5′-ATCCCCGTATTTCTGGTAGG-3′
GADPH	F:5’CCGGGAAACTGTGGCGTGATGG -3’
	R:5’AGGTGGAGGAGTGGGTGTCGCTGTT-3’
